# NAVIGATING HOSPICE CARE: PERSPECTIVES FROM OLDER BLACK AMERICANS

**DOI:** 10.1093/geroni/igae098.4244

**Published:** 2024-12-31

**Authors:** Channing Tate, Monica Perez-Jolles, Laura Schere, Tsion Shiferaw, Gwendolyn Mami, Daniel Matlock, Amy Huebschmann

**Affiliations:** University of Colorado AMC, Aurora, Colorado, United States; University of Colorado AMC, Aurora, Colorado, United States; University of Colorado AMC, Aurora, Colorado, United States; University of Colorado AMC, Aurora, Colorado, United States; Global Collaborations, LLC, Aurora, Colorado, United States; University of Colorado AMC, Aurora, Colorado, United States; University of Colorado AMC, Aurora, Colorado, United States

## Abstract

Misperceptions about hospice care are universally prevalent and disproportionately impact communities of color. Hospice remains an underutilized healthcare service, especially among older Black Americans. Potential cultural barriers to enrollment among Black Americans are important to identify. The study goal was to assess older Black adults’ perspectives of hospice care across four dimensions of hospice eligibility. A total of 144 participants responded to the written prompt, “In your own words, describe hospice care.” Written responses were evaluated to assess any misconceptions in their perspectives of hospice care. Participants were eligible if they were ≥65 years of age and self-identified as Black or African American. Participant ages ranged from 65 to 97 years (M =74.62, SD = 6.94), were predominately female (81%) and widowed (33%). Most participants accurately described hospice care/eligibility (80%), goals of hospice (89%), what services hospice provides (83%). However, only 39% of participants correctly identified locations of hospice services highlighting a potential barrier to hospice enrollment among older Black Americans. Additionally, some participants (8%) reported certain myths and conspiracies pertaining to hospice care, such as “hospice was created by the KKK”. These results highlight potential misunderstandings about hospice among older Black Americans that may serve as barriers to hospice enrollment and perpetuate inequities. These barriers go beyond hospice knowledge and include misconceptions and conspiracies rooted in medical mistrust and structural racism. The misconception that hospice care cannot be provided in the patients’ home is modifiable with interventions that use strategies tailored to support enhanced communication between patients and clinicians.

